# Combination of a Gellan Gum-Based Hydrogel With Cell Therapy for the Treatment of Cervical Spinal Cord Injury

**DOI:** 10.3389/fbioe.2020.00984

**Published:** 2020-08-26

**Authors:** Eduardo D. Gomes, Biswarup Ghosh, Rui Lima, Miguel Goulão, Tiago Moreira-Gomes, Joana Martins-Macedo, Mark W. Urban, Megan C. Wright, Jeffrey M. Gimble, Nuno Sousa, Nuno A. Silva, Angelo C. Lepore, António J. Salgado

**Affiliations:** ^1^Life and Health Sciences Research Institute (ICVS), School of Medicine, University of Minho, Braga, Portugal; ^2^ICVS/3B’s – PT Government Associate Laboratory, Guimarães, Portugal; ^3^Department of Neuroscience, Vickie and Jack Farber Institute for Neuroscience, Sidney Kimmel Medical College, Thomas Jefferson University, Philadelphia, PA, United States; ^4^Department of Biology, Arcadia University, Glenside, PA, United States; ^5^Center for Stem Cell Research and Regenerative Medicine, Tulane University, New Orleans, LA, United States

**Keywords:** cervical spinal cord injury, respiratory compromise, adipose tissue-derived stem/stromal cells, olfactory ensheathing cells, modified gellan gum hydrogels

## Abstract

Cervical spinal cord trauma represents more than half of the spinal cord injury (SCI) cases worldwide. Respiratory compromise, as well as severe limb motor deficits, are among the main consequences of cervical lesions. In the present work, a Gellan Gum (GG)-based hydrogel modified with GRGDS peptide, together with adipose tissue-derived stem/stromal cells (ASCs) and olfactory ensheathing cells (OECs), was used as a therapeutic strategy after a C2 hemisection SCI in rats. Hydrogel or cells alone, and a group without treatment, were also tested. Four weeks after injury, compound muscle action potentials (CMAPs) were performed to assess functional phrenic motor neuron (PhMN) innervation of the diaphragm; no differences were observed amongst groups, confirming that the PhMN pool located between C3 and C5 was not affected by the C2 injury or by the treatments. In the same line, the vast majority of diaphragmatic neuromuscular junctions remained intact. Five weeks post-injury, inspiratory bursting of the affected ipsilateral hemidiaphragm was evaluated through EMG recordings of dorsal, medial and ventral subregions of the muscle. All treatments significantly increased EMG amplitude at the ventral portion in comparison to untreated animals, but only the combinatorial group presented increased EMG amplitude at the medial portion of the hemidiaphragm. No differences were observed in forelimb motor function, neither in markers for axonal regrowth (neuronal tracers), astrogliosis (GFAP) and inflammatory cells (CD68). Moreover, using Von Frey testing of mechanical allodynia, it was possible to find a significant effect of the group combining hydrogel and cells on hypersensitivity; rats with a SCI displayed an increased response of the contralateral forelimb to a normally innocuous mechanical stimulus, but after treatment with the combinatorial therapy this behavior was reverted almost to the levels of uninjured controls. These results suggest that our therapeutic approach may have beneficial effects on both diaphragmatic recovery and sensory function.

## Introduction

Cervical spinal cord injuries (SCI) represent more than half of the SCI cases worldwide (Charsar et al., 2017). Traumatic lesions at cervical levels often result in respiratory compromise, due to damage to neural circuits controlling the diaphragm (Lane et al., 2008; Charsar et al., 2017). This circuitry is comprised of phrenic motor neurons (PhMN) located in the mid-cervical region of the spinal cord innervating the diaphragm, while in turn, this population is controlled by descending axonal input from rostral ventral respiratory group (rVRG) neurons located in the brainstem (Charsar et al., 2017). Despite the importance of this critical neural circuitry and the higher frequency of cervical lesions, a majority of pre-clinical studies have focused on thoracic lesions, which involve different affected circuitry and functions and eventually different responses to treatment.

Among the existing works in traumatic cervical SCI, cellular transplantation strategies have been designed to address the following purposes: replacing or inducing plasticity of neurons involved in respiratory circuits (Li et al., 2003; Alilain et al., 2011); replacing glial cell types (Li et al., 2015a) or local interneurons (Lee et al., 2014); providing trophic support (Gransee et al., 2015); and restoring neurotransmitter signaling (Li et al., 2015b).

Our group has developed a tissue engineering (TE) strategy for SCI repair, previously tested in a rat model of lumbar injuries (Gomes et al., 2016). This approach is based on the combination of adipose tissue-derived stem/stromal cells (ASCs) and olfactory ensheathing cells (OECs), together with a modified Gellan Gum (GG)-based hydrogel, which can be used both as a matrix for neural regrowth, and/or as a vehicle for cellular transplantation. ASCs and OECs represent two distinct cellular populations with complementary effects, as already demonstrated by our group (Silva et al., 2013a; Gomes et al., 2016, 2018). On one hand, OECs offer physical support and guidance for neurite outgrowth and elongation, mainly through direct cell–cell contact (Ramon-Cueto et al., 2000; Gomes et al., 2018). On the other hand, ASCs and their secreted factors are able to potentiate neurite formation and growth, promoting neuritogenesis, besides possessing immunomodulatory properties (Salgado et al., 2010; Lopez-Santalla et al., 2015; Gomes et al., 2018). Moreover, both cells can be obtained from autologous sources, increasing their potential application to the clinic. The GG hydrogel presents physical properties very similar to the spinal cord tissue and was previously modified with GRGDS motifs to increase cell adhesion (Silva et al., 2012a). This modification also led to improved morphology, viability and secretome properties of encapsulated cells (Silva et al., 2013b). In a lumbar SCI model, the combined therapy of hydrogel and cells led to significant locomotor improvements of the paralyzed hindlimbs, associated with a decreased inflammatory and astroglial response (Gomes et al., 2016).

Based on the abovementioned results, we determine whether the application of the same therapeutic strategy to a cervical hemisection injury could result in a beneficial outcome, modulating the local environment and possibly favoring neuronal preservation and/or inducing axon growth through the injury or in spared pathways. Toward this aim, following a C2 hemisection, ASCs and OECs encapsulated in the GG hydrogel were transplanted into the spinal cord lesion and functional and histological recovery was assessed.

## Materials and Methods

### ASCs and OECs Cultures

Human ASCs were isolated according to Dubois et al. (2008) in collaboration with LaCell LLC. Briefly, ASCs were isolated from human lipoaspirates obtained from consenting donors under an institutional review board approved protocol at LaCell LLC. These cells were cultured and maintained in α-MEM (Invitrogen, United States), with 10% Fetal Bovine Serum (FBS, Biochrom AG, Germany) and 1% antibiotic solution – penicillin/streptomycin (pen/strep; Invitrogen) at 37°C and 5% CO_2_ (v/v).

Olfactory ensheathing cells were isolated and cultured as previously described (Silva et al., 2012b). The animal care committees of the research institutes approved all the animal protocols in accordance with standardized animal care guidelines (Zutphen et al., 2001). Briefly, OECs were harvested from olfactory bulbs of neonatal (P5–P7) Wistar-Han rats. Upon dissection, the meninges and blood vessels were removed and the tissue was digested with collagenase type I (2.5 mg/ml, Sigma, United States) for 15 min at 37°C, with agitation. The digested tissue was mechanically dissociated with a 5 ml pipette and centrifuged at 175 G for 5 min. Then, the tissue was resuspended and subjected to a second mechanical dissociation using a P1000 micropipette. After a second centrifugation step, cells were resuspended and seeded on uncoated plates for two consecutive periods of 24 h. It is expected that most of the fibroblasts and astrocytes attach in the first and second periods, respectively. After this purification step, the remaining cells were seeded on fibronectin coated surfaces. Cells were cultured in DMEM/F12 (Invitrogen) with 10% FBS and 1% pen/strep solution at 37°C and 5% CO_2_ (v/v). OECs were additionally enriched with Bovine Pituitary Extract (5.36 μg/ml, Invitrogen) and Forskolin (1.4 μg/ml, Sigma).

### Hydrogel Preparation

The synthesis of GG-GRGDS hydrogel was performed according to the protocols described by Silva et al. (2012a). Briefly, GG (Sigma) was firstly dissolved in 2-(*N*-morpholino)ethanesulfonic acid (MES) buffer (100 mM, pH 5.5, Sigma) at 37°C. 4-(4,6-Dimethoxy-1,3,5-triazin-2-yl)-4-methylmorpholinium chloride (DMT-MM, Sigma) and furfurylamine (Acros Organics, Belgium) were then added in a 4:1 M ratio (of each reagent relative to the -COOH groups in GG) and stirred at 37°C for 48 h. The solution was then dialyzed (Mw cutoff 12–14 kDa, Spectrum Labs, United States) alternately against distilled water and phosphate buffered saline (PBS, 0.1M, pH 7.2) for 5 days. Finally, water was removed by lyophilization to obtain furan-modified GG (furan-GG) as a white powder. Immobilization of maleimide-modified GRGDS peptide (mal-GRGDS, Anaspec, United States) to furan-GG was performed via Diels-Alder chemistry between the maleimide functional group of the peptide with the furan group of the GG. Furan-GG was first dissolved in MES buffer at 37°C (1.2 mg/ml). Mal-GRGDS was then added in a 5:1 maleimide:furan molar ratio and vigorously stirred for 48 h. The solution was then dialyzed (Mw cutoff 12–14 kDa) alternately against distilled water and PBS for 5 days. Finally, the water was removed by lyophilization to obtain GRGDS-modified GG (GG-GRGDS) as a white powder.

### GG-GRGDS 3D Hydrogel Preparation

GG-GRGDS lyophilized powder was sterilized by exposure to UV lights for 15 min, a method previously used without affecting the material properties (Silva et al., 2013b). Then, GG-GRGDS was dissolved in ultrapure water, at 1% (w/v) concentration and heated at 40°C overnight, in order to obtain a homogenous solution. Before encapsulating the cells, CaCl_2_ at 0.3% (w/v) was added [to obtain a final concentration of 0.03% (w/v) of CaCl_2_ in solution] to enable the ionic crosslinking of the hydrogel.

### C2 Hemisection Injuries

#### Animals

As previously mentioned, all experimental procedures were approved either by the ICVS research committee and by the Thomas Jefferson University IACUC, being conducted in compliance with ARRIVE (Animal Research: Reporting of *In Vivo* Experiments) guidelines.

Ten weeks old female Wistar-Han rats (forelimb motor studies, Charles River, France) or twelve weeks old Sprague-Dawley (functional diaphragm studies, Taconic, United States), housed in light and temperature-controlled rooms and fed with standard diet, were used in the *in vivo* studies. Handling was performed for 3 days before the surgeries. Two sets of experiments were designed: the first one to assess diaphragm function and the second one dedicated to motor and sensory evaluation (Tables 1, 2).

**TABLE 1:** Experimental layout, with number of animals used per test, time points and rat breeds used in each set.

**Set**	**Set A – Forelimb motor studies**			**Set B – Functional diaphragm studies**		
**Rat breed**	**Wistar Han**			**Sprague Dawley**		
**Analysis**	**Test**	***n***	**Time point**	**Test**	***n***	**Time point**
	Staircase test	8–10/group	2 and 5 weeks	CMAPs	5–8/group	4 weeks
	Grooming test	8–9/group	3 weeks	NMJ morphology	3/group	5 weeks
	Von Frey test	7–10/group	4 weeks	EMGs	6–8/group	5 weeks
	GFAP and CD68	3/group	5 weeks	5-HT sprouting	4/group	5 weeks

**TABLE 2:** Total number of animals used per test, for each experimental group.

**Test**	**HS**	**GG-GRGDS**	**ASCs/OECs**	**GG-GRGDS + ASCs/OECs**
Staircase test	8	8	9	10
Grooming test	8	8	8	9
Von Frey test	7	8	7	10
GFAP and CD68	3	3	3	3
CMAPs	6	8	7	5
NMJs morphology	3	3	3	3
EMGs	6	8	7	8
5-HT sprouting	4	4	4	4

#### Cervical SCI and Treatments

All animals were anesthetized by intraperitoneal injection of a mixture (1.5:1) of ketamine (100 mg/ml, Imalgene/Merial, France) and medetomidine hydrochloride (1 mg/ml, Domitor/Pfizer, United States). Once anesthetized, the dorsal surface of the skin was shaved and disinfected with a 70% ethanol solution and topical iodine (Dynarex, Orangeburg, New York). Using a sterile #11 surgical blade (Electron Microscopy Sciences, Hatfield, PA, United States), a three-cm midline incision was made on the dorsal surface of skin and muscle, starting from the caudal portion of the occipital bone. Retractors were then used to expose the dorsal surface of the C2 and C3 vertebrae. Using rongeurs (Fine Science Tools, Foster City, CA, United States), remaining tissue was removed from the vertebrae and a laminectomy was performed to expose the spinal cord. The C2 and C3 dorsal roots were located, and a hemisection was performed at a location just caudal to the C2 root with a dissecting knife (Fine Science Tools, Foster City, CA, United States). To ensure a complete hemisection, a 30-gauge needle (BD Biosciences, San Jose, CA, United States) was passed through the injury several times. Within each set, animals were divided into four different groups according to the treatment/procedure: (1) animals subjected to SCI (hemisection) with no treatment (HS); (2) SCI animals treated with GG-GRGDS (GG-GRGDS); (3) SCI animals transplanted with cells (ASCs/OECs); (4) SCI animals treated with ASCs and OECs encapsulated in GG-GRGDS (GG-GRGDS + ASCs/OECs). The total animals per set and group is described in Table 3. Treatments were applied immediately after injury using a 30-gauge Hamilton syringe. The injection was performed manually, directly to the gap created by the lesion, at the slowest rate possible (around 1 min per injection). A total of 10 μl of hydrogel, cells or hydrogel with cells were injected per animal in the various groups. DMEM/F12 culture medium (OECs basal medium) was used as a vehicle for transplanted cells and injected as a control in non-treated animals. Rats treated with cells received a total of 200,000 cells (1:1 ASCs-OECs), either encapsulated in GG-GRGDS or in culture media. Following treatment, the dorsal muscle layers were sutured with 4–0 silk sutures (Covidien, Minneapolis, MN, United States), and the skin was closed with surgical staples (Fine Science Tools, Germany). The surface of the skin was treated with a topical iodine solution.

**TABLE 3:** Total number of animals used per group and in each experimental set.

	**Set A**	**Set B**	
HS	8	6	
GG-GRGDS	8	8	
ASCs/OECs	9	7	
GG-GRGDS + ASCs/OECs	10	8	
Total	35	29	64

#### Post-operative Care

Following SCI surgery and treatment, post-operative care was performed as previously described (Lima et al., 2017). Briefly, rats were kept under heat lamps and received subcutaneous injections of a solution containing vitamins (10 ml/Kg, Duphalyte/Pfizer, United States), 0.9% NaCl, the analgesic butorphanol (10 mg/ml, Butomidor/Richter Pharma AG, Austria), the antibiotic enrofloxacin (5 mg/ml, Baytril/Bayer, Germany), and atipamezole (5 mg/ml, Antisedan/Pfizer, United States) as a reversal agent. Bladder expression was checked to confirm that animals regained control. Then, for 5 days rats received daily subcutaneous injections of all the above-mentioned components except for atipamezole. Throughout the treatment and recovery period, animals were examined for symptoms of illness or potential reaction to the treatment. The diet was enriched and the food was presented to the rats on the cage floor.

### rVRG Axon Tracing

Three weeks after injury, animals were anesthetized as described in cervical injuries and subjected to intra-brainstem injections of AAV2-mCherry, as described previously (Goulao et al., 2019). A midline incision was made at the base of the cranium using a sterile #11 blade. After deflection of the muscle and the C1/cranium ligament, the bone covering a portion of the brainstem was removed. Using a Hamilton Gastight Syringe (Hamilton, Reno, NV, United States) with a 33-gauge needle, 0.3 μl of virus was injected 2 mm lateral to the right (ipsilateral rVRG tracing), 1 mm rostral and 2.6 mm ventral to the brainstem obex, using a stereotaxic apparatus (Kopf Instruments, Tujunga, CA, United States) and an UltraMicroPump (World Precision Instruments, Sarasota, FL, United States). The needle was left in place for 5 min before careful retrieval from the medulla. Postoperative care was given as described for C2 hemisection injuries. Spinal cord sections were later analyzed for mCherry labeling using a Zeiss Imager M2 upright microscope with Metamorph Software.

### Compound Muscle Action Potential (CMAP) Recordings

Four weeks post-surgery, rats were anesthetized with isoflurane (Piramal Healthcare, Bethlehem, PA, United States) at a concentration of 3.0–3.5% diluted in oxygen. Animals were placed supine and the region just below the rib cage was shaved and cleaned with 70% ethanol. Phrenic nerve conduction studies were performed with stimulation at the neck via near nerve needle electrodes placed along the phrenic nerve. A reference electrode was placed on the shaved surface of the right costal region. The phrenic nerve was stimulated with a single burst at 6 mV (amplitude) for a 0.5 ms duration. Each animal was stimulated between 10 and 20 times to ensure reproducibility, and recordings were averaged for analysis. Animals were daily followed for any signs of distress in response to this procedure. ADI Powerlab 8/30 stimulator and BioAMPamplifier (ADInstruments, Colorado Springs, CO, United States) were used for both stimulation and recording, and Scope 3.5.6 software (ADInstruments, Colorado Springs, CO, United States) was used for subsequent data analysis. An additional control animal without lesion (laminectomy only) was used as an example of a normal CMAP recording.

### Electromyography (EMG) Recordings

Five weeks post-surgery and immediately before sacrifice, animals were anesthetized with isoflurane at a concentration of 3.0–3.5% diluted in oxygen. All animals had fully recovered from CMAP recordings, with no signs of distress. Once deeply anesthetized, a laparotomy was performed to expose the right hemi-diaphragm. Bipolar electrodes spaced 3 mm apart were placed for recording in three separate sub-regions of the hemi-diaphragm: dorsal, medial, and ventral. Electrodes were always placed at the endplate band at all recording locations. Two recordings were averaged over a 2-min time frame for each animal, and peak amplitude, burst duration and frequency were taken. Using LabChart 7 software (AD Instruments, Colorado Springs, CO, United States), the EMG signal was amplified and filtered through a band-pass filter (50–3000 Hz). Following recordings, animals were immediately euthanized with a triple-dose of ketamine/xylazine/acepromazine and the spinal cord and diaphragm were collected after transcardial perfusion with 4% paraformaldehyde. As in CMAP recordings, an additional control animal without lesion (laminectomy only) was used as an example of a normal EMG recording.

### Limb Motor Assessment

#### Staircase Test

The staircase (also called skilled paw reaching test) was performed with double staircase boxes (Campden Instruments, Lafayette, IN, United States), as previously described (Campos et al., 2013). The shape and dimensions of the boxes were similar to the ones described by Montoya et al. (1991). The apparatus consists of a clear chamber with a hinged lid that was developed to assess independent forelimb use in skilled reaching and grasping tasks. A narrow compartment, with a central platform running along its length, is connected to this chamber. The removable double staircase with seven steps on each side can be inserted in the space between the platform and the box walls. Five pellets were placed into each well of the double staircase apparatus. On the first day, the rats were familiarized with the test and pellets were freely available at random positions for 10 min. During the test session, animals were kept inside the box and had 15 min to reach, retrieve, and eat food pellets present on the steps. All sessions were performed at the same time of day under food-restriction. After each test interval, animals were removed from the staircase boxes and the uneaten pellets were counted. During the first five test days, rats were presented with pellets on both sides of the staircase, while in the sixth and seventh day, pellets were placed only on one side, alternating sides from 1 day to the other (forced choice paradigm). The staircase test was performed during the second- and fifth-weeks post-injury. As the main outputs, eating scores were calculated. It considers the ratio between the number of pellets eaten and the total number of pellets available.

#### Grooming Test

Three weeks after injury grooming behavior was evaluated for all rats. A soft gauze with fresh tap water was applied to the rats’ back head to induce grooming. Then the rats were placed in a glass cylinder and their behavior was filmed. After two complete cycles of grooming (starting by licking the paws and ending by cleaning behind the ears) the rats were placed back in their cages. Later, both forelimbs were scored using a scale varying from 0 (unable to touch the nose) up to 5 (reach the back of their ears), adapting a protocol from Bertelli and Mira (1993).

### Von Frey Analysis

The Von Frey test was performed at 4 weeks post-injury, as described in Guimaraes et al. (2019). Animals were placed in an elevated grid and left to acclimatize to the experimental conditions for 5 min. Mechanical allodynia was then assessed using the up-and-down method (Chaplan et al., 1994) as described previously (Sotiropoulos et al., 2014). Briefly, the sural dermatome of the contralateral limbs (fore- and hindimb) was probed with a series of von Frey calibrated monofilaments: 15.0, 8.0, 6.0, 4.0, 2.0, 1.0, 0.6, and 0.4 g (North Coast Medical Inc., United States). Starting with the 2.0 g filament, the test would advance upward if no response was elicited (=0) or downward if a brisk withdrawn of the limb was produced (=X) until 6 measurements were obtained around the threshold point according to the model developed by Dixon (Dixon, 1980). Paw movements, associated with locomotion or weight shifting, were not counted as a response. The 50% response threshold was then calculated using the following formula:

$$50\%\,g\,\_\,t\,h\,r\,e\,s\,h\,o\,l\,d=\frac{\left(10^{X\,f+K.\delta }\right)}{10000}$$

where X_f_ = value (in log units) of the final von Frey filament; k = tabular value corresponding to pattern of positive and negative responses [X and 0 sequence; consult (Chaplan et al., 1994)]; δ = mean difference (in log units) between stimuli (0.224). If no response was obtained up to maximal force (15.0 g) or conversely, if all filaments elicited a response down to the minimal force (0.4 g), the values 15 and 0.25 were assumed as the 50% withdrawal threshold, respectively. In this experiment, a total of three animals without injury were used as controls.

### Histological Characterization

In both experimental sets, animals were sacrificed 5 weeks post-injury/treatment.

#### Neuromuscular Junction (NMJ) Analysis

Four animals per group were used for NMJ analysis. Rats were euthanized with a mixture of ketamine/xylazine/acepromazine. Animals were placed supine; two incisions were made into the skin and underlying muscle starting from the xyphoid process and extending laterally along the rib cage to expose the right hemidiaphragm. The right hemi-diaphragm was excised using spring scissors (Fine Science Tools, Foster City, CA, United States), stretched flat and pinned down on silicon-coated 10 cm dishes, and washed with PBS (Gibco, Pittsburgh, PA, United States). Next, a 20-min fixation in 4% paraformaldehyde (PFA, Electron Microscopy Sciences, Hatfield, PA, United States) was performed, followed by several washes in PBS. After washing, superficial fascia was carefully removed from the surface of the diaphragm with Dumont #5 Forceps (Fine Science Tools, Foster City, CA, United States).

Whole-mount immunohistochemistry was performed, as described previously (Wright and Son, 2007). Diaphragms were rinsed 3× in PBS and then incubated in 0.1M glycine for 30 min. Following glycine incubation, α-bungarotoxin conjugated to Alexa Fluor 555 at 1:200 (Life Technologies, Waltham, MA, United States) was used to label post-synaptic nicotinic acetylcholine receptors for 15 min and then washed 3× in PBS. Ice-cold methanol was then added to the diaphragms for 5 min and then washed 3× in PBS. Diaphragms were then blocked for 1 h at room temperature in a solution of 2% bovine serum albumin and 0.2% Triton X-100 diluted in PBS (this solution was used for both primary and secondary antibody dilutions). Primary antibodies were added overnight at 4°C: pre-synaptic vesicle marker anti-SV2 at 1:10 (Developmental Studies Hybridoma Bank, Iowa City, IA, United States), neurofilament marker anti-SMI-312 at 1:1000 (Covance, Greenfield, IN, United States). On the following morning, the diaphragms were washed 3 × in blocking solution, and secondary antibody solution was then added for 1 h at room temperature: FITC anti-mouse IgG secondary (Jackson ImmunoResearch Laboratories, West Grove, PA, United States; 1:100), followed by washing 3× in PBS. Diaphragms were mounted with Vectashield mounting medium (Vector Laboratories, Burlingame, CA, United States), coverslips were added, and slides were stored at −20°C.

We morphologically evaluated NMJ innervation by quantifying three phenotypes at individual synapses: intact NMJs, completely denervated NMJs, and partially denervated NMJs. We identified intact NMJs by complete overlap of the pre-synaptic axon labeling with the post-synaptic α-bungarotoxin labeling. We defined completely denervated NMJs by total absence of overlap between pre- and post-synaptic marker labeling. Partially denervated NMJs showed some overlap of pre- and post-synaptic labeling; however, this overlap was not complete as observed with intact NMJs. For each muscle/animal, we quantified 200–300 NMJs across the entire hemi-diaphragm, and we expressed the data for each phenotype as the percentage of total NMJs. Whole-mounted diaphragms were imaged on a FluoView FV1000 confocal microscope (Olympus, Center Valley, PA, United States). We conducted NMJ analysis on ipsilateral hemi-diaphragm because previous published work showed no denervation or sprouting in contralateral hemi-diaphragm after cervical injury (Nicaise et al., 2012a,b).

#### Spinal Cord Tissue Processing

Five weeks post-injury, a rough dissection of the spine and spinal cord was performed in PFA perfused animals, centered on the site of hemisection and the tissues were additionally fixed in 4% PFA overnight. A more detailed dissection of the spinal cord was then conducted and the tissues were carefully placed on a solution of saccharose at 30% (w/v). After 24 h, 2.5–3 cm length of spinal cord tissues, centered on the lesion, were involved in frozen section medium (Neg-50, Thermo Scientific, United States), frozen with liquid nitrogen and stored at −20°C. Later on, longitudinal cross sections of 20 μm thickness were performed using a Leica CM1900 cryostat.

#### Immunohistochemistry

Frozen longitudinal sections of cervical spinal cord were air-dried and washed three times (5 min each) with PBS. Samples were then incubated in blocking solution (5% normal goat serum and 0.4% Triton X-100 diluted in PBS) for 1 h at room temperature. Sections were incubated overnight at 4°C with primary antibodies in blocking solution. The following primary antibodies were used: polyclonal rabbit 5-HT antibody (Immunostar, Hudson, WI, United States), polyclonal rabbit anti-Glial Fibrillary Acidic Protein (GFAP, Dako, Denmark) and monoclonal mouse anti-CD68 (Merck Millipore, MA, United States). Sections were then washed with PBS (three washes, 5 min each) and incubated with the following secondary antibodies conjugated to Alexa fluorophores (Invitrogen) in blocking solution for 1 h at room temperature: AF488 goat anti-rabbit (Jackson ImmunoResearch Laboratories, West Grove, PA, United States) for 5-HT, AF488 goat anti-rabbit (Invitrogen) for GFAP and AF594 goat anti-mouse (Invitrogen) for CD68. After washing with PBS (three washes, 5 min each), sections were coverslipped. For 5-HT staining, images were acquired with a Zeiss Imager M2 upright microscope, and MetaMorph software was used to quantify 5-HT immunostaining. For quantification of CD68+ reactive macrophages/monocytes, and GFAP+ cells, tissue sections were imaged on an Olympus IX81 inverted microscope. The total area of CD68 and GFAP expressing cells was quantified in 4–6 slices per animal.

### Statistics

All statistical analyses were performed using GraphPad Prism version 5.00 for Windows (GraphPad Software, United States). Differences among groups were assessed by one-way ANOVA test or by the two-way ANOVA test followed by the Bonferroni *post hoc* test, in data with a normal distribution. Data without a normal distribution or with a small number of samples were analyzed with the non-parametric test Kruskal–Wallis followed by the Dunn’s multiple comparison test. A *p*-value of ≤0.05 (95% confidence level) was set as the criteria for statistical significance, for all the analyses performed.

## Results

### Functional and Morphological Innervation of the Ipsilateral Hemi-Diaphragm

**FIGURE 1:**
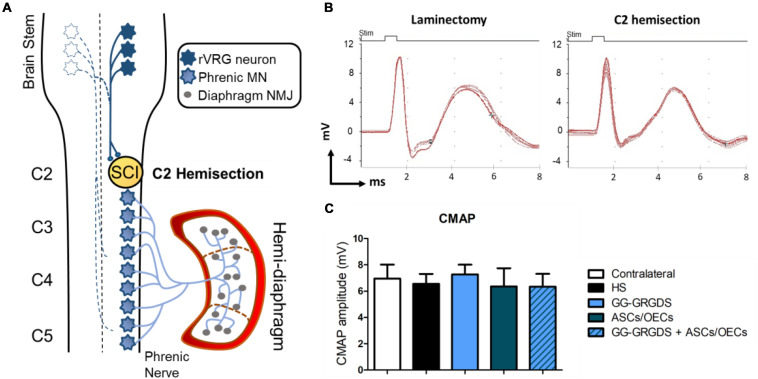
Cervical hemisection injury at C2 level **(A)** does not affect CMAP amplitudes **(B,C)** analyzed at the ipsilateral hemi-diaphragm. **(A)** Schematic representation of the neuronal pathways controlling the hemi-diaphragm. Contralateral rVRG neurons are represented as white-filled stars. **(B)** Examples of the CMAP signals detected for a rat without SCI (laminectomy only) and a C2 hemisected rat. **(C)** Quantification of the average CMAP amplitude (mV) in the four treated groups and in the contralateral side of the injury. Data is presented as mean ± SD (*n* = 5–8 per group).

**FIGURE 2:**
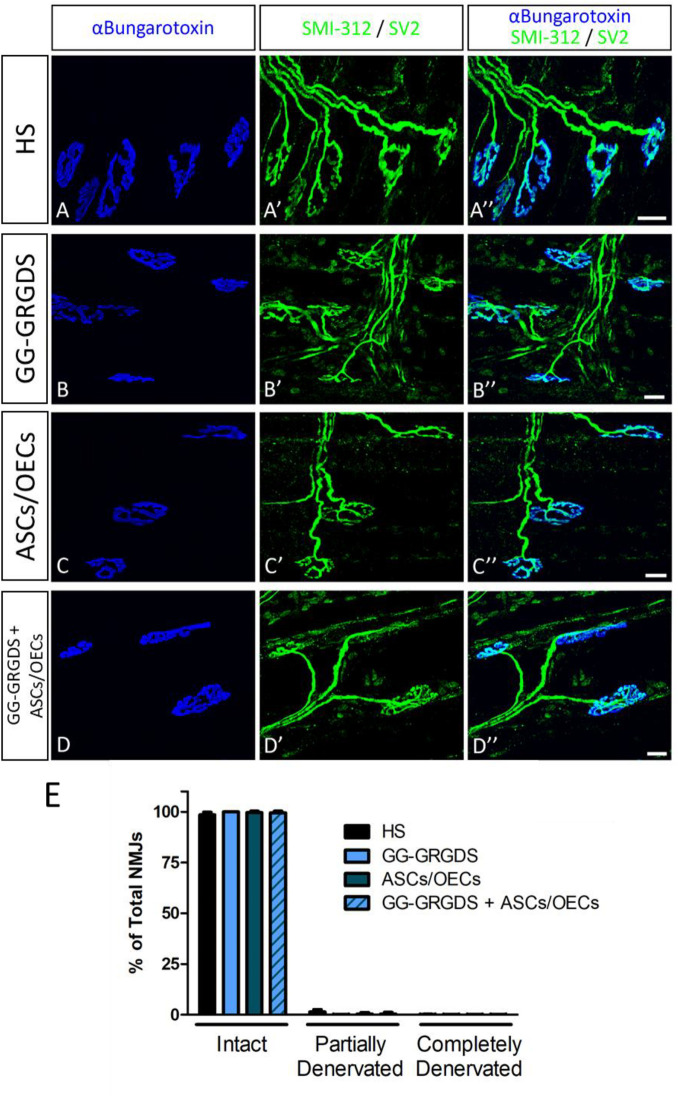
NMJ morphology in the ipsilateral hemi-diaphragm. No significant alterations were seen among groups and most of the NMJs were intact. Nicotinic acetylcholine receptors stained with α-bungarotoxin (in blue, **A–D**) and pre-synaptic terminals stained with SMI-312 and SV2 (in green, **A’–D’**). **(A”–D”)**: merged images. **(E)** Quantification of intact, partially-denervated and completely-denervated NMJs at the ipsilateral hemi-diaphragm. Data is presented as median ± IQR (*n* = 3 per group).

Lesions at the C2 spinal cord level are not expected to directly affect the PhMN pool located between C3 and C5. Therefore, in order to confirm the functional innervation of the ipsilateral hemi-diaphragm, CMAPs were recorded after supramaximal stimulation of the phrenic nerve. No significant alterations were observed among groups regarding CMAP amplitudes (Figures 1A,B), even in comparison to the non-lesioned contralateral hemi-diaphragm (Figure 1B). Moreover, after histological assessment of NMJ morphologies in the ipsilateral hemi-diaphragm, no differences were seen in the percentage of intact, partially denervated or completely denervated NMJs (Figure 2). The majority of NMJs analyzed were intact (almost 100%), demonstrating that the injury did not affect diaphragmatic morphological innervation. In both CMAP and NMJ assessments, the treatments did not influence the results obtained.

### Ipsilateral Hemi-Diaphragm Function Assessment

**FIGURE 3:**
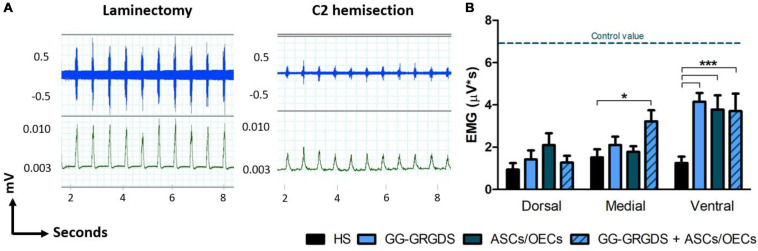
EMG recovery in the ipsilateral hemi-diaphragm, 5 weeks after C2 cervical hemisection injury. **(A)** Non-treated rats (right graph) present a reduced EMG activity (dorsal recordings represented) in comparison to non-lesioned rats (only subjected to a laminectomy, left graph), revealing a normal pattern of EMG activity. Blue traces are the raw EMG signals (top) whereas green representative traces are the integrated EMG signals (bottom). **(B)** Treatments with hydrogel, cells or both led to significant recovery of integrated EMG amplitude at the ventral portion of the ipsilateral hemi-diaphragm, while in the medial portion only the combinatorial treatment provided significant improvement. No differences were seen among groups at the dorsal portion. Data is presented as mean ± SEM (*n* = 6–8 per group); *p < 0.05, ***p < 0.001.

Five weeks after injury, diaphragmatic function was assessed through EMG analysis. After recordings at three different subregions of the hemi-diaphragm ipsilateral to injury, it was observed a clear reduction in EMG amplitudes following SCI (Figures 3A,B). At the ventral portion of the hemi-diaphragm, all treatments induced a significant recovery of EMG amplitude. However, it should be highlighted that at the medial portion only the combination of hydrogel and cells was capable of promoting a significant improvement (Figure 3B). This result suggests a beneficial effect for the conjugation of hydrogel and cells.

### Serotonergic Innervation of the Ipsilateral Cervical Spinal Cord

**FIGURE 4:**
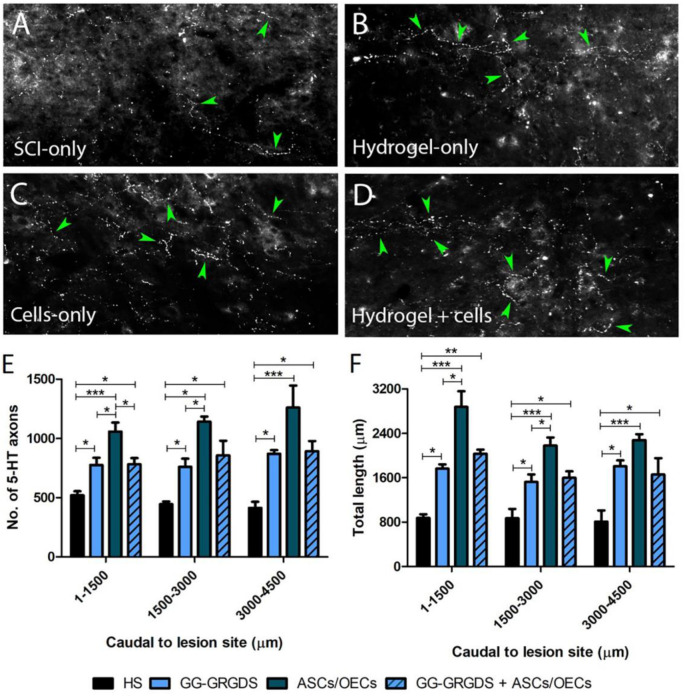
Serotonergic axon sprouting caudal to the ipsilateral lesion site. **(A–D)** Representative confocal images of 5-HT fibers in the caudal cervical spinal cord. **(E,F)** 5-HT axon numbers and total length. Data is presented as mean ± SD (*n* = 4 per group).

Serotonergic descending input plays an important role in the excitability of the spinal cord motor neurons, including PhMNs. Therefore, 5-HT axon sprouting was assessed at the ipsilateral lesioned side of the spinal cord specifically within the PhMN pool. Interestingly, all treatments induced an increase in the number and length of 5-HT fibers caudal to the lesion site in the ipsilateral C3-C5 ventral horn (Figure 4), with the cells-only and combined treatment inducing the highest levels of serotonergic fiber sprouting.

### Forelimb Motor Evaluation

**FIGURE 5:**
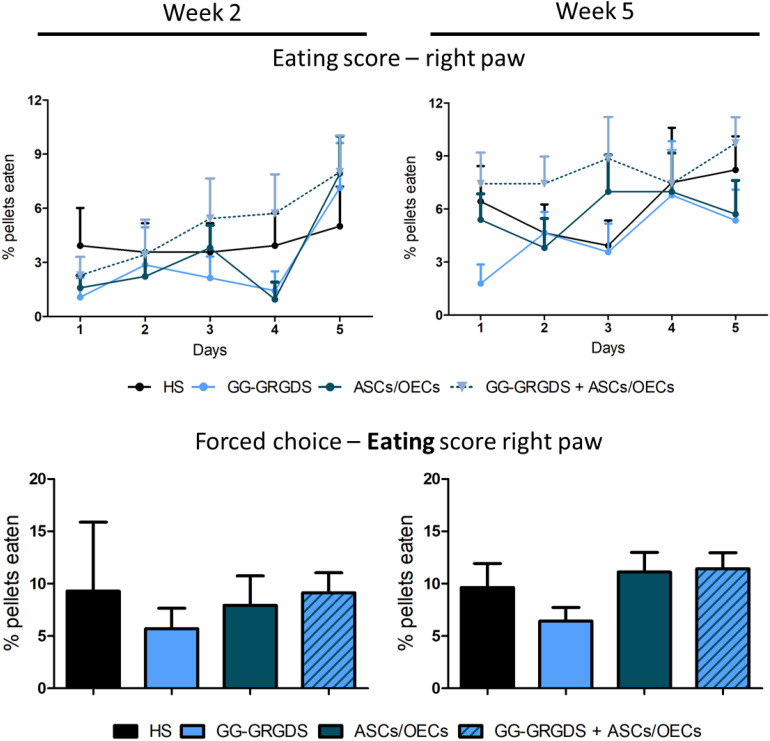
Staircase test for the right paw, performed at **2** and **5** weeks after injury. There are no significant differences among groups in none of the time points evaluated, for both the staircase test **(upper graphs)** and the forced choice task of the test **(bottom graphs)**. Data is presented as mean ± SEM (*n* = 8–10 per group).

Motor impairment of the forelimbs is another significant consequence of cervical SCI. In the C2 hemisection, only the right forelimb was expected to be affected. In order to evaluate the recovery of motor function, two different motor behavior paradigms were performed. The first one was the staircase test, in which rats under food restriction are assessed for their fine motor skills, such as reaching and grasping abilities, to retrieve and eat a sugared pellet. At 2 and 5 weeks after injury, rats were subjected to the test, where during the first 5 days they could retrieve pellets from both right and left sides and in the last 2 days, pellets were only available on the left or on the right side (forced choice, Figure 5). In both modalities of the test, no significant differences were observed among groups for the eating score, either in the learning phase (Figure 5, top graphs) or in the forced choice paradigm (Figure 5, bottom graphs).

**FIGURE 6:**
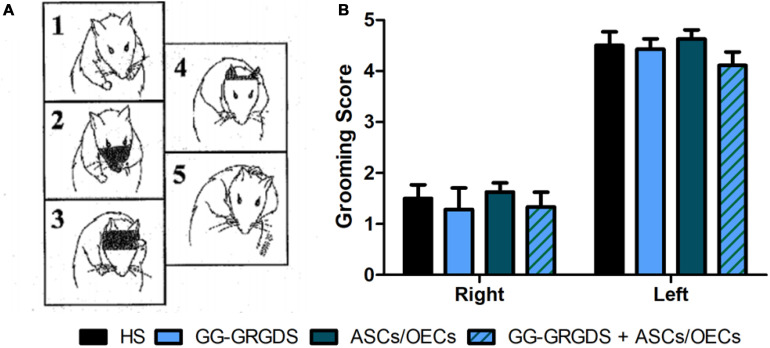
Grooming test performed at 3 weeks after injury. **(A)** Representative images of the grooming scale behavior, with 1 meaning limited forepaw movement and 5 normal forepaw function. **(B)** No differences were seen in grooming capacity among groups, for both right and left forelimbs. As expected, the lesioned right paw presents deficits in grooming ability. Data is presented as mean ± SEM (*n* = 8 per group).

The second motor paradigm used was the grooming test. Grooming is a natural behavior in rodents that can be used to assess forelimb movement capacity. Using a pre-defined scale, where 1 means limited movements, while 5 represents a normal movement with complete grooming cycles, rats were evaluated at 3 weeks post-injury (Figure 6). The movement capacity of the right forelimb was significantly affected in all rats; however, there were no differences among groups. There were also no differences across treatment groups for the left, contralateral forelimb.

Overall, these motor paradigms reinforce the idea that the injury was correctly performed, but treatments were unable to induce any motor recovery.

### Sensory Function After Injury

**FIGURE 7:**
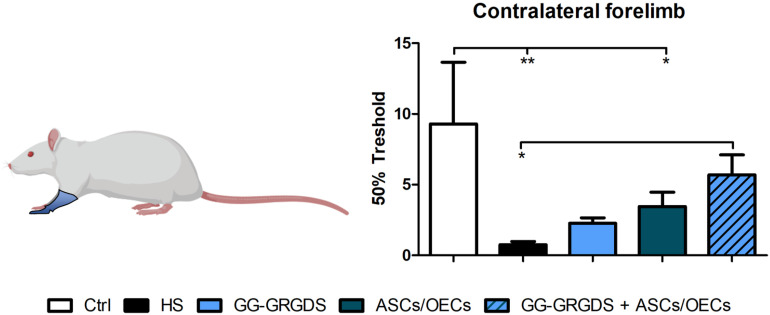
Von Frey test performed at 4 weeks after injury. Forelimb sensory responses to Von Frey filaments were assessed and quantified. The combinatorial treatment led to a recovered sensory phenotype for the contralateral forelimb. Non-lesioned rats were used as a control. Data is presented as mean ± SEM (*n* = 8 per group); *p < 0.05, **p < 0.01.

A common outcome following SCI is sensory dysfunction. With the objective of evaluating the response to mechanical stimuli, rats were subjected to the Von Frey test 4 weeks post-lesion. Following C2 cervical injury, it was observed a marked increase in sensitivity of the contralateral limbs (Figure 7), responding to a stimulus usually innocuous to non-injured rats. However, after treatments, all treated groups demonstrated recovery from the hypersensitivity observed in the left hindpaw, while very interestingly, in the forepaw, only the combinatorial strategy induced significant recovery of sensory function to levels similar to uninjured controls (Figure 7).

## Discussion

Cervical SCI pre-clinical research is of great clinical relevance since lesions at this level account for more than half of SCI cases every year (Singh et al., 2014). TE approaches to cervical lesions have already been employed with some success (Liu et al., 2017; Geissler et al., 2018). For instance, Geissler et al. (2018) transplanted neural progenitor cells on a collagen-based hydrogel leading to functional recovery of rats with unilateral cervical contusion injuries. In another study, Liu et al. (2017) combined alginate hydrogels seeded with Schwann cells with BDNF viral delivery, in a C5 lateral hemisection model in rats. They showed that serotonergic and other descending axons could be traced throughout the scaffolds. Ghosh et al. (2018) have recently demonstrated that local BDNF delivery using an engineered hydrogel significantly enhanced diaphragmatic respiratory function.

In our case, the combination of ASCs and OECs with the peptide-grafted GG hydrogel has led to relevant functional and histological improvements in other models of SCI (Gomes et al., 2016). After a hemisection injury at the thoracic level, the administration of cellular transplants of ASCs/OECs was sufficient to induce a significant recovery of locomotor function, assessed by the BBB test, together with a marked decrease in inflammatory cells close to the lesion site (Gomes et al., 2018). On the other hand, in a more aggressive hemisection lumbar injury model, the combination of ASCs/OECs with GG-GRGDS hydrogel was the only treatment capable of promoting functional locomotor improvements, associated with similar decreases in inflammatory cells. In addition, the combined treatment reduced the levels of astrocytes close to the injury and preserved the levels of neurofilament-positive cells. Importantly, the number of ASCs that survived transplantation was higher when cells were transplanted within the hydrogel matrix (Gomes et al., 2016). Taking these results into account, the objective of this study was to ascertain if the same strategy could induce therapeutic efficacy in a cervical model of SCI.

While most clinical cases of SCI are contusive in nature, the C2 hemisection is a powerful model to investigate mechanisms of axon regeneration and sprouting as it creates a very precise and restricted injury, as well as complete ablation of defined axonal pathways. C2 hemisection interrupts the input of rVRG neurons from the brainstem, to PhMNs located around C3-C5 segments resulting in paralysis of the ipsilateral hemidiaphragm. In this sense, as a first experiment, the functionality and morphology of diaphragmatic innervation by PhMNs were analyzed (Figures 1, 2). As expected, there were no alterations in CMAP amplitudes, and no significant morphological denervation of NMJs of the hemidiaphragm. This was already seen in other recent works from the group using C2 cervical hemisections (Urban et al., 2018). This happens because the PhMN pool responsible for diaphragm innervation is not directly affected by the lesion, maintaining a response when activated and preserving overall NMJs structures. However, since they lose supraspinal input from the brainstem (rVRG neurons), they stop controlling diaphragmatic function. This loss of function is then reflected in the EMG recordings of the lesioned hemi-diaphragm (Figure 3). In the present work, different regions were analyzed independently, to dissect more closely the effects observed. Even though no differences were seen among groups at the dorsal portion of the diaphragm, medial and ventral portions registered significant improvements. In the ventral portion all treatments provided significant recovery of function, while in the medial portion, only the combinatorial treatment resulted in a significantly increased EMG signal. It is known that rostral spinal cord segments of the PhMN pool (C3) innervate the ventral region of the costal and crural areas of the diaphragm muscle, while more caudal segments innervate more dorsal portions (C5) (Fogarty et al., 2018). This anatomical characteristic might justify the higher improvement observed in ventral portions, as the treatments (hydrogel and/or cells) are applied at C2 level, possibly indicating a proximity-based effect. The combined treatment was effectively the only one capable of inducing partial recovery of EMG activity at the medial portion of the hemi-diaphragm, indicative of a more potent beneficial effect, in comparison to single treatment of hydrogel or cells. The hydrogel alone also induced partial recovery of function at the ventral portion. In this sense, the physical support provided by GG-GRGDS could also be in part responsible for the recovery of function. As in the previous work (Gomes et al., 2016), the hydrogel was degraded with time, not being found at 5 weeks post-implantation. Yet, its confinement to the injury site was visually confirmed post-injection. Following the analysis of the spinal cord tissue, the most significant results were related with serotonergic innervation of the ipsilateral hemidiaphragm (Figure 4). Serotonin is synthesized in different populations of brainstem neurons and plays a crucial role in modulating motor function (Ghosh and Pearse, 2014). We observed a significant increase in all treatment groups, in the number and total length of 5-HT axons, in comparison to non-treated rats. This increased sprouting of 5-HT axons might partially account for the recovery observed in diaphragmatic function. The transplantation of OECs for instance, has been associated with increased regeneration of serotonergic fibers through different lesions, such as thoracic transection (Ramon-Cueto et al., 1998; Lu et al., 2001). The group transplanted with cells only presented the most significant recovery of 5-HT fibers, however this was not correlated with EMG signals. Therefore, other mechanisms might also be influencing diaphragmatic function, which could justify the improved EMG bursting in the combinatorial group.

Regarding limb motor function analysis, none of the therapeutic strategies employed impacted motor recovery. The motor behavior paradigms used address general forelimb movements (grooming test) but also skilled and fine detailed movements (staircase test). The specificity of the cervical neuronal circuitry might explain the different results observed between cervical and thoracic/lumbar studies. Forelimb motor function is highly dependent on supraspinal inputs, while thoracic and lumbar-derived movements rely significantly on local circuitries, such as central pattern generator (CPG) activity (Filli et al., 2011). Long-distance CST projections are difficult to regenerate, and we observed no regrowth of rVRG axons into and through the lesion site (Supplementary Figure S1). No differences were also observed in astrogliosis and inflammatory levels, as measured by the total area of GFAP and CD68 positive cells (Supplementary Figure S2).

Interestingly, sensorial perception of the contralateral forelimb was also normalized following the combined treatment (Figure 7). The sensorial fibers are also affected following SCI, with patients frequently developing chronic pain (Siddall et al., 1999). In this sense, the recovery observed is also very important, for the establishment of therapies that could improve a rescue of different systems, often considered more important than locomotion by the patients (Anderson, 2004). The explanation for this recovery is still elusive, although chronic pain and sensorial mechanisms have been associated with inflammation (Detloff et al., 2008), and our strategy has demonstrated immuno-modulatory properties in other two injury models (Gomes et al., 2016, 2018).

Cells from non-autologous sources were used in this study, both human ASCs and rat pup derived OECs. The use of autologous cells could have impacted differently the results observed, as it is expected that cell rejection rates are less significant. Still, we previously demonstrated that human ASCs can be integrated into the spinal cord tissue and survive for more than 8 weeks (Gomes et al., 2016, 2018), which favors the potential application of human ASCs to the clinics. In fact, both ASCs and OECs have already been tested independently in human clinical trials, demonstrating to be safe (Mackay-Sim et al., 2008; Hur et al., 2016). Hence, their conjugation might represent a valuable step in SCI management, as it can ameliorate the results obtained from each cell type individually.

## Conclusion

Cervical SCI are the frequent in humans, therefore, there is a need for more studies focusing on traumatic lesions affecting this spinal cord region. TE approaches such as the one presented here are promising, as they can address multiple targets, increasing the chances for functional recovery following an injury. In our work, the combination of GG-GRGDS hydrogel with ASCs/OECs led to an increased diaphragmatic activity together with a partial reestablishment of sensory function compromised by the injury. This is of the utmost relevance, as respiratory compromise and chronic pain are two of the main concerns of SCI patients. The specific mechanism(s) by which this therapeutic strategy exerts its effects may be related – at least in part – to an increase in specific serotonergic fiber sprouting in the ipsilateral caudal spinal cord. This strategy opens a window for improvement of a critical condition such as cervical SCI.

## Data Availability Statement

The datasets generated for this study are available on request to the corresponding author.

## Ethics Statement

Human lipoaspirates obtained from consenting donors under an institutional review board approved protocol at LaCell LLC.

## Author Contributions

EG and BG contributed to the experimental design, data collection, analysis and interpretation, and drafting of the manuscript. RL, MG, TM-G, JM-M, MU, and MW contributed to the data collection, analysis, and interpretation. JG and NS provided the technical knowledge and materials for the experiments. NAS, AL, and AS interpreted the data, supervised the work, and revised the manuscript. All the authors approved the final version of the manuscript.

## Conflict of Interest

The authors declare that the research was conducted in the absence of any commercial or financial relationships that could be construed as a potential conflict of interest.

## Supplementary Material

FIGURE S1 mCherry-labeled rVrg axons detected at the intact rostral spinal cord tissue, 5 weeks post-injury. Only a limited number of axons entered the lesion site, not growing through the lesion. No differences were observed amongst groups. **(A)** Rats without treatment; **(B)** Gg-Grgds-only treated rats; **(C)** Ascs/Oecs-only treated rats; **(D)** Gg-Grgds + Ascs/Oecs treated animals.

FIGURE S2 Quantification of the total area occupied by Gfap+ **(A)** and Cd68+ **(B)** cells. No differences were observed amongst groups. Data is presented as mean ± Sem (*n* = 3 per group).
