# Supplementary figures and images for: Combination of a Gellan Gum-Based Hydrogel With Cell Therapy for the Treatment of Cervical Spinal Cord Injury
Source: Front Bioeng Biotechnol. 2020 Aug 26;8:984. doi: 10.3389/fbioe.2020.00984 (PMC7479129; doi:10.3389/fbioe.2020.00984)

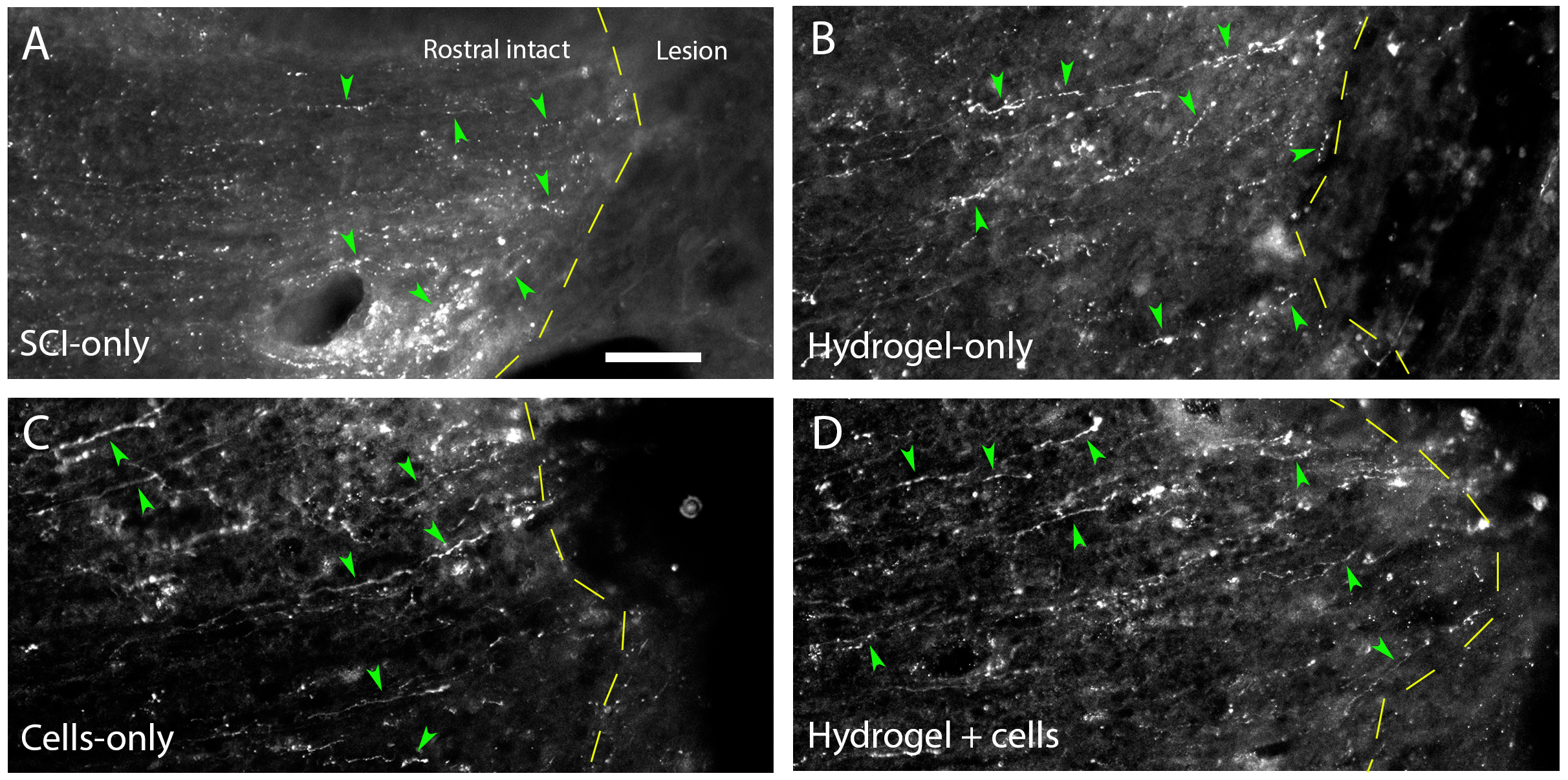

Supplement: FIGURE S1 — mCherry-labeled rVrg axons detected at the intact rostral spinal cord tissue, 5 weeks post-injury. Only a limited number of axons entered the lesion site, not growing through the lesion. No differences were observed amongst groups. (A) Rats without treatment; (B) Gg-Grgds-only treated rats; (C) Ascs/Oecs-only treated rats; (D) Gg-Grgds + Ascs/Oecs treated animals. [file Image_1.tif]

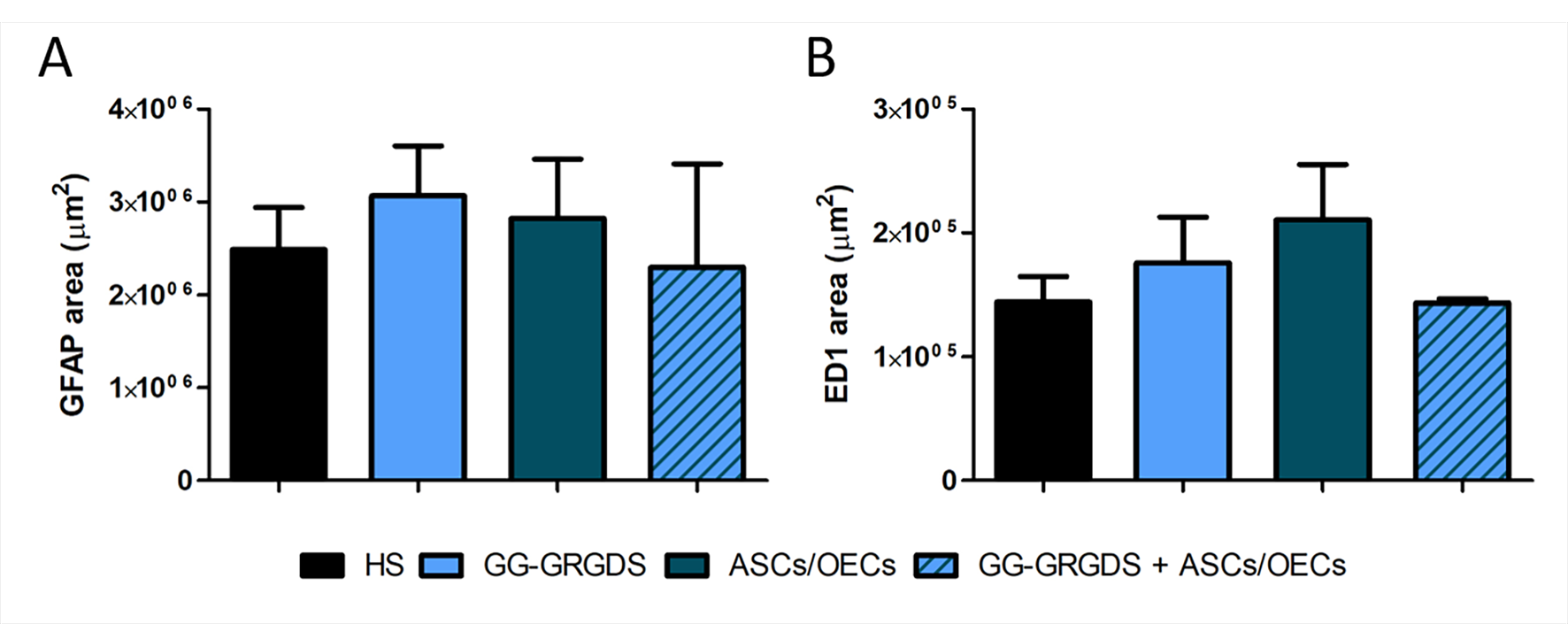

Supplement: FIGURE S2 — Quantification of the total area occupied by Gfap+ (A) and Cd68+ (B) cells. No differences were observed amongst groups. Data is presented as mean ± Sem (n = 3 per group). [file Image_2.tif]
